# Persimmon Leaves (*Diospyros kaki*) Extract Enhances the Viability of Human Corneal Endothelial Cells by Improving Na^+^-K^+^-ATPase Activity

**DOI:** 10.3390/ph15010072

**Published:** 2022-01-06

**Authors:** Ramsha Afzal, Hyung Bin Hwang

**Affiliations:** Department of Ophthalmology, Incheon St. Mary Hospital, College of Medicine, The Catholic University of Korea, 58 Dongsu-ro, Bupyeong-gu, Incheon 403-720, Korea; arosho175@gmail.com

**Keywords:** Na^+^/K^+^-ATPase, human corneal endothelial cells, ethanol extract of *Diospyros kaki*, cornea, cell viability, enzymatic activity, staurosporine, ouabain, persimmon leaves

## Abstract

The Na^+^/K^+^-ATPase, present in the basolateral membrane of human corneal endothelial cells (HCECs), is known to play an important role for corneal transparency. Na^+^/K^+^-ATPase dysfunction is one of the major causes of corneal decompensation. The ethanol extract of *Diospyros kaki* (EEDK) has been reported to increase corneal cell viability. Thus, we treated HCECs with EEDK and studied its effects on HCECs survival and Na^+^/K^+^-ATPase against cytotoxic drugs like staurosporine (ST) and ouabain (OU). Firstly, survival assays, (MTT assay and live dead-imaging) showed that decreased HCECs viability by ST and OU was significantly recovered by EEDK co-treatment. Secondly, Na^+^/K^+^-ATPase activity assays revealed that EEDK enhanced Na^+^/K^+^-ATPase enzymatic activity (* *p* < 0.01) with/without ST and OU. Finally, Na^+^/K^+^-ATPase expression analysis (Western Blot and confocal microscopy) demonstrated that EEDK treatment with/without ST and OU facilitates Na^+^/K^+^-ATPase expression in HCECs. Taken together, our findings led us to the conclusion that EEDK might aid HCECs survival in vitro by increasing the activity and expression of Na^+^/K^+^-ATPase enzyme. Since Na^+^/K^+^-ATPase activity is important to maintain cellular function of HCECs, we suggest that EEDK can be a potential effective agent against corneal edema and related corneal disorders.

## 1. Introduction

The cornea is a transparent outer layer of the eye that covers the entire front of the eye and focuses the light allowing for clear vision. It consists of several layers of cells like the epithelium, Bowman’s layer, stroma, Descemet’s membrane, and endothelial layer. The corneal epithelium (the outer-most layer) is a well-characterized self-renewing layer with stem cells at its peripheral areas. The corneal stroma makes up 90% of cornea and its hydration plays a major role in maintaining corneal transparency. The posterior side of the corneal stroma is lined by Descemet’s membrane and the innermost monolayer membrane of cornea is endothelium. The corneal endothelial cells are composed of hexagonal, squamous cells lining the posterior cornea. Apoptosis or necrosis of human corneal endothelial cells (HCECs) may lead to corneal edema and decompensation [1].

HCECs maintain a barrier between the cornea and aqueous humor [2]. The Na^+^/K^+^-ATPase, present in the basolateral membrane of HCECs, functions both as an active pump and as a barrier against fluid incursion into the cornea. Mainly, Na^+^/K^+^-ATPase maintains adequate corneal hydration while allowing adequate nutrients delivery to the stroma and epithelium. Na^+^/K^+^-ATPase activity keeps the clear cornea and its disruption results in corneal edema and visual loss [3,4,5]. Since HCECs do not proliferate in humans, it is important to prevent damage to HCECs and to maintain its function [6,7]. The Na^+^/K^+^-ATPase regulates pump function by extruding Na^+^ (depolarization) and restoring K^+^ (repolarization) via an active-transport mechanism [8]. HCECs express different Na^+^/K^+^-ATPase isoforms, including metabolically active α_1_ and α_3_ isoforms [9,10]. Some neurodegenerative diseases [11], as well as corneal endothelial dysfunction, chronic inflammation, and other cellular diseases, are linked to declined Na^+^/K^+^-ATPase pump activity and numbers [12,13,14].

Persimmons (*Diospyros kaki*) are mostly grown in East Asian countries (South Korea, Japan, and China) [15] and have been used as herbal medicine for centuries. The ethanol extract of *D. Kaki* leaves (EEDK) has been studied as a drug against certain ocular disorders [16]. EEDK originated from persimmon leaves contain antioxidant, anti-inflammatory, and anti-hypertensive properties [17,18]. EEDK is rich in flavonoids [19], which have been proved to exhibit anti-inflammatory activities in various cells and animal models [20,21]. Previously, EEDK was reported to have anti-inflammatory effects on a dry-eye model [22,23,24] and it is known that corneal epithelial cells are vulnerable to any stress. So, we hypothesized that EEDK might help HCECs to improve their viability and Na^+^/K^+^-ATPase activity under stress conditions.

In this study, we used cytotoxic controls, staurosporine (ST) and ouabain (OU), and studied their effects on HCECs and Na^+^/K^+^-ATPase in the presence of EEDK. ST is a nonspecific serine-threonine kinase (PKC) inhibitor [25] that reduces the Na^+^/K^+^-ATPase pump current [26] while OU is a cardiotonic steroid that binds to Na^+^/K^+^-ATPase, causing conformational changes [27]. This study aims to evaluate the effect of EEDK on Na^+^/K^+^-ATPase in HCECs through cell-viability assays, live/dead cell imaging, protein quantification, confocal-microscopy, and enzymatic assay. We found that EEDK has the potential to be an effective agent to help HCECs viability.

## 2. Results

### 2.1. EEDK Increased HCECs Viability

**Figure 1:**
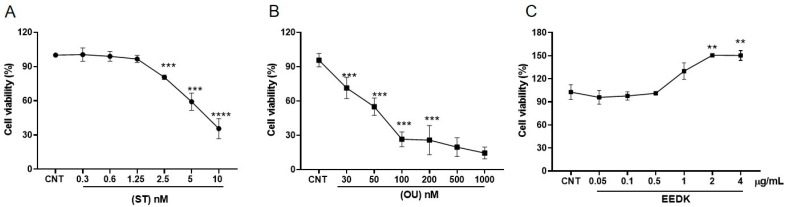

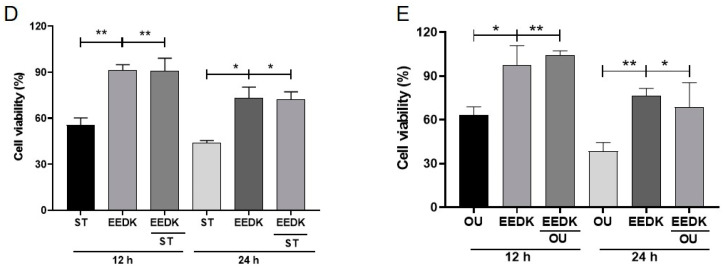
Optimization of cytotoxic drugs concentration and viability of HCECs under the influence of ST and OU with or without EEDK. HCECs were exposed to (**A**) ST and (**B**) OU, and (**C**) levels of EEDK were also optimized. EEDK co-treatment with (**D**) ST and (**E**) OU. Data are presented as the mean ± standard error mean (SEM) of values from four representative experiments. * *p* < 0.05, ** *p* < 0.01, *** *p* < 0.001, **** *p* < 0.0001 for the indicated comparisons (one-way ANOVA with Dunnett’s multiple comparison test).

To analyze the viability of HCECs under stress conditions and EEDK treatment, a series of MTT assays were performed to optimize the concentration of ST, OU, and EEDK (Figure 1A–C). We selected ST and OU concentrations, which showed a ~50% decrease in HCECs viability (5 nM, 50 nM respectively) (Figure 1A,B). For EEDK, we observed that a 2 µg/mL final concentration showed a significant increase (~60%, ** *p* < 0.01) in HCECs viability (Figure 1C). The co-treatments of EEDK with ST/OU on HCECs at different time-lapses (Figure 1D,E) showed that EEDK enhanced the lifespan of cells by 30% after 12 h and 20% after 24 h (Figure 1D). Similarly, EEDK co-treatment with OU elevated cell viability by 40% after 12 h and 30% after 24 h (Figure 1E). Our viability assay data suggested that EEDK treatment enables HCECs to survive against cytotoxic drugs (ST and OU).

### 2.2. Fluorescence Microscopy for Live/Dead Cells

**Figure 2:**
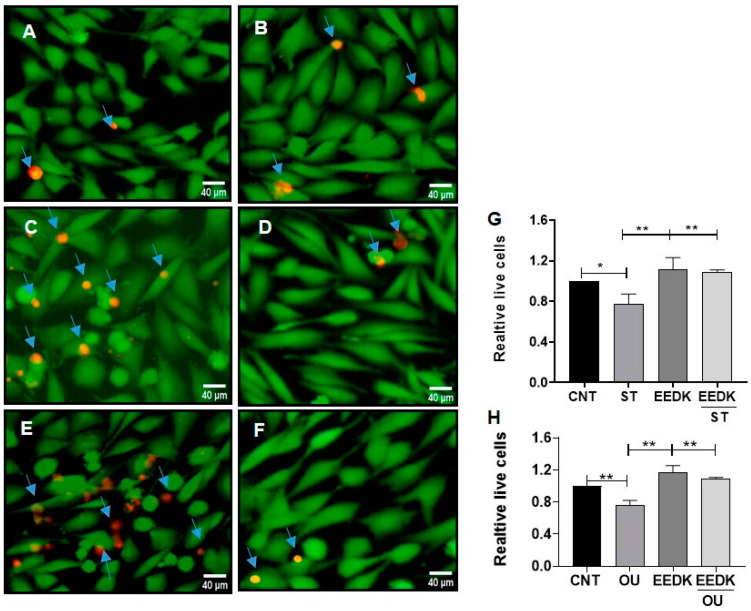
Live/dead imaging of HCECs exposed to cytotoxic drugs (ST and OU) with or without EEDK co-treatment. (**A**,**B**) Representative images of cells exposed to controls (**A**; no drug treatment, **B**; EEDK treated cells) respectively. (**C**,**D**) Representative images of cells exposed to ST (**C**) and its co-treatment with EEDK 2 µg/mL (**D**). (**E**,**F**) Representative images of cells exposed to OU (E) and its co-treatment with EEDK 2 µg/mL (**F**). (**G**,**H**) Representative graphs for the ST and OU groups, respectively. Data are presented as the standard error mean ± SEM of values from four representative experiments. * *p* < 0.05, ** *p* < 0.01 for the indicated comparisons (one-way ANOVA with Tukey’s multiple comparison test).

Figure 2 demonstrates images of cells in a live/dead assay combining green and red fluorescence (live and dead cells respectively). Figure 2A,B are the representative images of non-treated and EEDK-treated cells (controls), respectively. Our results indicated that co-treatment of EEDK with cytotoxic drugs had a positive effect on the viability of HCECs as compared to the cells exposed to ST (Figure 2C,D) and OU alone (Figure 2E,F). Figure 2G,H are the illustrative graphs of the ST group and OU group respectively.

### 2.3. Enhanced Na^+^/K^+^-ATPase Enzymatic Activity by EEDK

**Figure 3:**
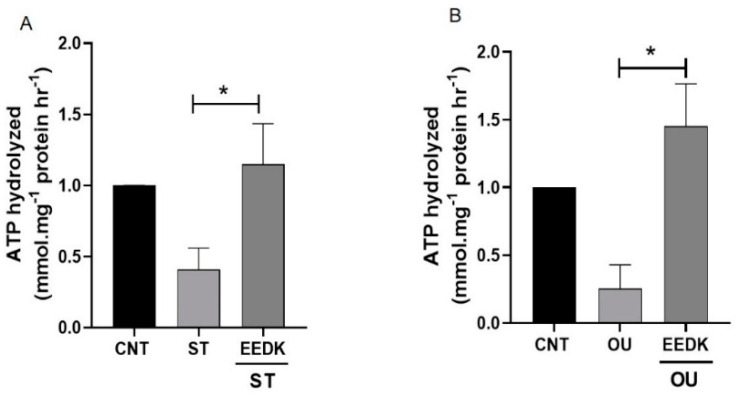
Effects of EEDK on Na^+^/K^+^-ATPase activity in cultured HCECs. (**A**) Cells were treated with ST 5 nM and ST+ EEDK 2 µg/mL for 24 h and then assayed for Na^+^/K^+^-ATPase activity. (**B**) Cells were treated with 50 nM of OU with or without 2 µg/mL EEDK. Non-treated cells were used as control in both cases. Data are presented as the standard error mean ± SEM of values from three representative experiments. * *p* < 0.05 for the indicated comparisons (one-way ANOVA with Tukey’s multiple comparison test).

To analyze Na^+^/K^+^-ATPase activity, ATP hydrolysis reactions were analyzed as described previously [28]. It was seen that ATPase activity was reduced markedly in ST and OU-treated groups while EEDK co-treatment (with ST and OU) showed a significant increase (Figure 3A,B) in enzymatic activity. These findings showed that EEDK treatment has the potential to effectively stabilize the Na^+^/K^+^-ATPase enzymatic activity.

### 2.4. EEDK Co-Treatment Increased the Na^+^/K^+^-ATPase Expression in HCECs

**Figure 4:**
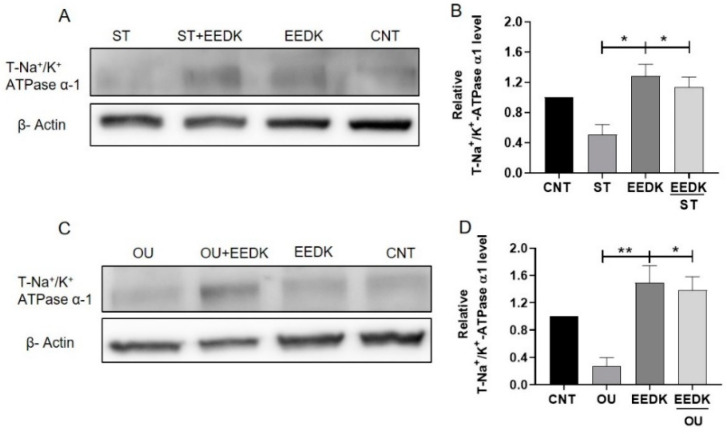
Protein expression of total-Na^+^/K^+^-ATPase α1 subunit by western blot analysis. (**A**) Representative signals of expression. Top: Na^+^/K^+^-ATPase α_1_-subunit. Bottom: β-Actin, the relative intensity of each band to β-actin was measured by a densitometer as the expression of Na^+^/K^+^-ATPase α_1_-subunit. (**B**) Representative graph of 4A. Cells were exposed to ST 5 nM and ST with EEDK 2 µg/mL. Only EEDK and non-treated cells were used as controls after 24 h of treatment cells were assayed to analyze the expression level of Na^+^/K^+^-ATPase α_1_. (**C**) Representative signals of expression. Top: Na^+^/K^+^-ATPase α_1_-subunit. Bottom: β-Actin. (**D**) Representative graph of 4C. Cells were incubated with OU 50 nM with and without EEDK 2 µg/mL. Non-treated cells and only EEDK treated cells represent controls. Data are presented as the mean ± SEM of values from three experiments, * *p* < 0.05, ** *p* < 0.01 for the indicated comparisons (one-way ANOVA with Tukey’s multiple comparison test).

To determine Na^+^/K^+^-ATPase protein expression, HCECs were exposed to cytotoxic drugs (ST and OU) with/without EEDK for 24 h. total Na^+^/K^+^-ATPase α_1_ protein expression levels were measured using western blot analysis. The results for total Na^+^/K^+^-ATPase (T-Na^+^/K^+^-ATPase α_1_) indicated that co-treatment of ST with EEDK tends to increase the protein expression level in HCECs (Figure 4A,B). A similar trend was observed in the groups co-treated with OU and EEDK (Figure 4C,D). The increase in the T-Na^+^/K^+^-ATPase α1 level was significant in both ST and OU groups.

### 2.5. Localization of Na^+^/K^+^-ATPase in Basolateral Membrane

To analyze the effect of EEDK on Na^+^/K^+^-ATPase located in the basolateral membrane of HCECs, cells were treated with drugs as described previously and analyzed via confocal microscopy. The results followed the same pattern as seen in the previous Western blot analysis.

## 3. Discussion

In this study, we proved that EEDK treatment increases HCECs viability, enzyme activity and expression of the Na^+^/K^+^-ATPase in cultured HCECs with/without cytotoxic agents. Both HCECs and the corneal Na^+^/K^+^-ATPase are responsible for maintaining corneal transparency and are vulnerable to toxic and inflammatory substances. So, cellular and ocular surface damages occur as a result of the decrease in number of cells or dysfunction of Na^+^/K^+^-ATPase [29].

*D. kaki* leaves have been studied to have multiple nutritional and medicinal benefits. EEDK has been considered an effective drug against glaucoma and it enhances retinal cell survival by decreasing apoptotic proteins [29] and inflammation [22,30]. So, we hypothesized that EEDK might facilitate HCECs viability and improve Na^+^/K^+^-ATPase functions (enzyme activity and protein expression). Secondly, we expected that EEDK treatment would compensate for the cytotoxic effects of drugs (ST and OU), which included a decrease in HCECs population (in vitro) and Na^+^/K^+^-ATPase function. So, based on these hypotheses, we investigated the effect of EEDK on HCECs against cytotoxic drugs (ST and OU).

Here, we used ST because its treatment decreased the Protein Kinase C (PKC) activity [30] that in turn interfere with the Na^+^/K^+^-ATPase activity by direct phosphorylation of serine18 [31]. Whereas OU inhibits the growth of cells through the activation of protein kinases [32,33]. OU activates Src kinase, which causes cell growth arrest by increasing the expression of the cell cycle inhibitor (p21) [34].

Our cell viability assays suggested that EEDK significantly increases the viability of HCECs as compared to ST- and OU-treated cells (Figure 1). Similarly, live-dead imaging data exhibited the same trend (Figure 2). We found that cell viability and live/dead imaging assay comply with our hypotheses that EEDK helps HCECs to grow healthy even in stress conditions. Then, the next step was to find out the effect of HCECs on Na^+^/K^+^-ATPase.

The Na^+^/K^+^-ATPase is the largest protein complex in the family of P-type cation pumps and α1 isoform are known to play a more generalized role among four α-subunits (α1, α2, α3, and α4) [9]. The Na^+^/K^+^-ATPase α1 activity is an important determinant of HCECs’ ability to maintain the water content of the corneal stroma and uncontrolled water flow by the downregulation of Na^+^/K^+^-ATPase can induce corneal edema [35,36]. That is, the Na^+^/K^+^-ATPase activity maintains the hydration of the cornea [37,38].

**Figure 5:**
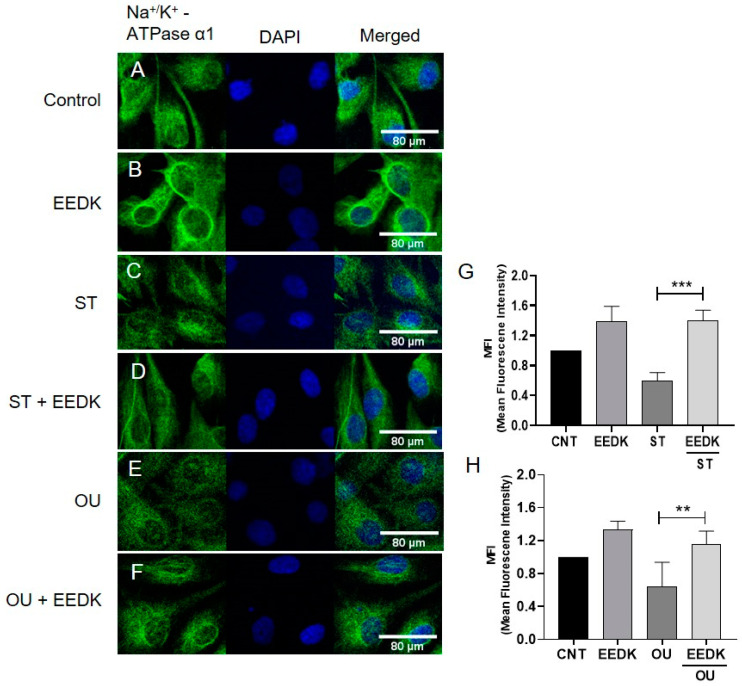
Confocal microscopy for Na^+^/K^+^-ATPase α1 cell surface expression in cultured HCECs. HCECs seeded on coverslips and incubated with ST or OU with/without EEDK for 24 h and then stained with Na^+^/K^+^-ATPase antibody (green fluorescence, membranous) and DAPI (blue). (**A**) Un-treated HCECs (CNT), (**B**) HCECs treated with 2 µg/mL EEDK, (**C**) cells treated with 5 nM ST, (**D**) HCECs undergone co-treatment of ST and EEDK, (**E**) HCECs with 50 nM OU, (**F**) cells co-treated with OU and EEDK, and (**G**,**H**) graphical representation of ST and OU group respectively. Results are expressed as the mean ± SEM of three independent experiments. ** *p* < 0.01, *** *p* < 0.001 (one-way ANOVA with Dunnett’s multiple comparison).

We observed the elevated enzymatic activity of Na^+^/K^+^-ATPase in EEDK-treated cells. The decreased Na^+^/K^+^-ATPase activity by ST can be recovered by EEDK co-treatment (Figure 3A). We presume that this increased Na^+^/K^+^-ATPase activity by EEDK appears to be mediated by PKC. Along with that, the lowered Na^+^/K^+^-ATPase activity by OU treatment was also recovered by EEDK co-treatment (Figure 3B). But the effect of EEDK on induction of Src and p21 needs experimental verifications. Additionally, the protein expression assays (western blot and confocal microscopy) revealed that ST and OU decreased the Na^+^/K^+^-ATPase α1-subunit expression, which was significantly revived by the co-treatment of EEDK (Figure 4 and Figure 5). Our results suggest that EEDK can increase HCECs viability by targeting its key enzyme Na^+^/K^+^-ATPase.

In this study, we tried to unveil many aspects of EEDK, but there are still some limitations. Firstly, our results demonstrate that EEDK raises the enzymatic activity and expression of Na^+^/K^+^-ATPase. However, the exact mechanism through which EEDK influences the activity of the Na^+^/K^+^-ATPase is unknown, and more research is needed to uncover the relevant pathways. Secondly, EEDK has been shown to have anti-inflammatory [20,21,22,23,24] as well as antioxidant effects [17,24]. As can be concluded from our study, EEDK enables HCECs to maintain Na^+^/K^+^-ATPase activity, which is responsible for lessening the stromal inflammation [12,13,14] and it most likely follows an anti-inflammatory mechanism here. But the experiments related to the anti-inflammatory pathway are not performed here. Pro-inflammatory cytokines (interleukins and tumor necrosis factor families) expression by EEDK treatment should be analyzed in the future study. Even though we proved that EEDK may have a significant impact on the Na^+^/K^+^-ATPase in HCECs, the current study is only conducted with HCECs in vitro.

In conclusion, our data suggest that EEDK increases the viability of HCECs against cytotoxic agents and even enhances the Na^+^/K^+^-ATPase activity and expression. To our knowledge, this is the first study to demonstrate the positive effect of EEDK on Na^+^/K^+^-ATPase and HCECs. Through further studies, we can expect the efficacy of EEDK as a potential drug to corneal endothelial dysfunction.

## 4. Materials and Methods

### 4.1. Chemicals

Staurosporine, Ouabain octahydrate, 3-(4,5-Dimethylthiazol-2-yl)-2,5-Diphenyltetrazolium Bromide (MTT) reagent, and Dimethyl sulfoxide (DMSO) were purchased from Sigma Aldrich (St. Louis, MO, USA). Chemicals used to grow HCECs were all purchased from Applied Biological Materials (ABM Inc., Toronto, ON, Canada) that includes Prigrow-I medium, Fetal bovine serum (FBS), human insulin, human transferrin, sodium selenite, hydrocortisone, β-estradiol, human vascular endothelial growth factor 165aa, human endothelial growth factor, heparin, l-glutamine, and penicillin/streptomycin solution, and applied cell extracellular matrix (ECM). Radioimmunoprecipitation assay (RIPA) buffer was purchased from GenDepot (Houston, TX, USA); all western blot buffers were purchased from Biosaesang (Gyeonggi-do, Korea); ammonium molybdate (Biomol Green) and phosphate standards were obtained from Biomol Research Laboratories (Plymouth Meeting, Montgomery, PA, USA); BCA protein assay kits and enhanced chemiluminescence (ECL) western blotting substrate were obtained from Thermo Fisher Scientific (Waltham, MA, USA); anti-Na^+^/K^+^-ATPase α_1_ antibody was obtained from Santa Cruz Biotechnology (Santa Cruz, CA, USA); and anti-β-actin, anti-mouse, and anti-rabbit antibodies were purchased from Cell Signaling Technology (Danvers, MA, USA). The live/dead cell staining kit was purchased from Biomax (Seoul, Korea). Ethanol extract of Persimmon leaves (EEDK) was a gift from the Natural Products Research Center, Korea Institute of Science and Technology (KIST).

### 4.2. Cell Culture

Immortalized HCECs were purchased from Applied Biological Materials (Richmond, BC, Canada). ECM-coated plates were used to grow HCECs for better adhesion and used for all experiments. Cells were cultured in a humidified 5% CO_2_ atmosphere at 37 °C in Prigrow-I medium. The complete growth medium included 10% fetal bovine serum, 5 mg/L of human insulin, 10 µg/mL of human transferrin, 3 ng/mL of sodium selenite, 10 nM of hydrocortisone, 10 nM of β-estradiol, 10 ng/mL of human vascular endothelial growth factor 165aa, 10 ng/mL of human endothelial growth factor, 10 ng/mL of heparin, 1% l-glutamine, and 1% penicillin/streptomycin solution. Experiments were performed when cells reached approximately 70–80% confluent. All the experiments were repeated at a minimum of thrice.

### 4.3. Concentrations Optimization

The HCECs were treated with serially diluted drugs to determine the toxicity and to optimize the parameters (ST: 0.3–10 nM; OU: 30–1000 nM, EEDK: 0.05–4 µg/mL). In the case of cytotoxic drugs (ST and OU), the concentrations that resulted in a ~50% reduction of cells viability were selected for further experiments. For ST, we used 5 nM and for OU 50 nM. For EEDK we used a broad range of concentrations to optimize the conditions (data not shown). Here, we presented the concentrations near optimal concentration (EEDK: 2 µg/mL). We also treated HCECs with combinations of any of two compounds (ST and OU) with EEDK. Here, untreated HCECs were considered as positive control cells.

### 4.4. Cell Viability (MTT) Assay

A total of 2 × 10^4^ cells/100 μL cells were seeded in 96-well plates for 24 h followed by serum starvation for 4 h and then subjected to drug treatments for 20 h (total 24 h). Ten microliters of MTT was added at a 1/10 ratio (final concentration, 0.5 mg/mL) for 4 h at 37 °C. The media was removed, and the reduced MTT (blue formazan) was solubilized with DMSO. After 15 min, the optical density at 570 nm (OD_570_) was measured.

### 4.5. EZ-Live/Dead Imaging

Calcein-AM [39] and propidium iodide [40] were used to label live and dead cells respectively. Cells were grown in a manner that, after 24 h, form a monolayer in 24-well plates and is then exposed to drugs (ST and OU) with or without EEDK for 24 h. After washing three times with 1× phosphate-buffered saline (PBS), cells were incubated with calcein-AM and propidium iodide for 45 min. Images were taken under a fluorescence microscope with excitation at 490 and 545 nm

We looked at regions that appeared to be confluent and had been labeled with calcein-AM and propidium iodide. To compute the mean numbers of live/dead cells per field, 10 randomly selected fields under the microscope were assessed per coverslip, and two coverslips were analyzed for each of three independent cultures. These counts were done three times in total. One field represented 0.385 mm^2^ of culture area, while one coverslip had a total area of 7.1 mm^2^, consequently, each field represented about 5% of the overall coverslip area, as assessed by a stage micrometer. Live and dead cells were counted using ImageJ software (NIH, Bethesda, MD, USA). The percentages of live and dead cells were determined from each treatment group. All groups were normalized by the control to find out the relative live cells.

### 4.6. Measurement of Na^+^/K^+^-ATPase Activity

To measure enzymatic activity, we followed the protocol described previously [28,41]. Briefly, the cells media was removed and ultrapure distilled water (150 µL) was added to each well. The plates were then placed in liquid nitrogen for 10 sec before adding 150 µL of solution containing 80 mM of histidine, 20 mM of KCl, 6 mM of MgCl_2_, 2 mM of EGTA, Alamethicin (2 µg/mL), 30 µM of digitonin, and 200 mM of NaCl at 7.4 pH. Ten microliters of 30 mM of ouabain (final concentration, 1 mM) or vehicle were added to duplicate wells, and the plate was incubated at 37 °C for 30 min. The reaction mixtures were incubated for a further 30 min at 37 °C after the addition of 10 µL of 300 mM ATP (final concentration, 10 mM). The ATP hydrolysis reaction was stopped by pouring 75 µL of 50% trichloroacetic acid into each well. At RT, the contents of wells were centrifuged at 3000 rpm for 10 min.

The resulting supernatants were diluted 50 times with ultrapure distilled water, and portions (50 µL) of the diluted samples were added to tubes containing 100 µL of ammonium molybdate reagent (Biomol Green; Biomol Research Laboratories) for phosphate content determination by measuring absorbance at 640 nm following the manufacturer’s instructions. The phosphate solutions ranging from 0 to 40 M were used as standards. The Na^+^/K^+^-ATPase activity was calculated as the difference in the ATPase activity of cells in the presence vs. absence of OU and was expressed as millimoles of ATP hydrolyzed per milligram of protein per hour.

### 4.7. Western Blot Analysis of the Na^+^/K^+^-ATPase α_1_ Subunit

HCECs were grown on six-well plates and treated with drugs as described earlier. After 24 h, the culture medium was removed and after washing with PBS, cells were lysed with RIPA buffer and total protein was extracted, which was measured by BCA assay. A total of 10 μg of protein for each sample was subjected to 8% SDS-PAGE and transferred onto polyvinylidene difluoride (PVDF) membranes. After blocking with 5% skim-milk in 0.1% Tween-20 in TBS (TBST), the membranes were incubated with anti-Na^+^/K^+^-ATPase α_1_ antibody (1:2000 dilution with TBST), or an anti-β-actin antibody (1:3000 dilution with TBST) overnight at 4 °C. Followed by washing three times with TBST, secondary antibodies were applied for 1 h separately at RT. Positive immunoreactivity was visualized using ECL western blotting substrate.

### 4.8. Confocal Microscopy

HCECs were seeded and cultured on coverslips in 24 well plates. After washing with PBS, HCECs were fixed with 4% paraformaldehyde at RT for 15min. After washing, cells were blocked with PBS containing 3% BSA (Biosesang, Seongnam, Korea) and 0.3% Triton X-100 (Biosesang) for 20 min. Slides were incubated with the primary antibody (anti-Na^+^/K^+^-ATPase α_1_ antibody) in a 1:500 ratio for 1 h at RT. After repeated washing, the slides were incubated with Alexa FluorTM 594 conjugated goat anti-rabbit IgG (Invitrogen, Carlsbad, CA, USA) (1:1000) in a blocking solution for 30 min at RT. After subsequent washing, slides were examined under a LSM 700 confocal microscope (Carl Zeiss Meditec, Inc., Dublin, CA, USA). The intensities of the HCECs were determined using ImageJ software (NIH, Bethesda, MD, USA). All groups were normalized by the control to find out the men fluorescence intensities (MFI).

### 4.9. Statistical Analysis

Here, we analyzed our data using GraphPad prism 8.0 software (GraphPad Software, San Diego, CA, USA). All data reported in this study were expressed as mean ± standard error of mean (SEM). One-way analysis of variance (one-way ANOVA) with Dunnett’s test or Tukey’s test was performed to compare differences among more than two groups. In all analyses, * *p* < 0.05, ** *p* < 0.01, and *** *p* < 0.005 were taken to indicate statistical significance.

## 5. Conclusions

Our data led us to the conclusions that EEDK effectively enhanced the HCECs survival by increasing both the activity and expression of Na^+^/K^+^-ATPase. To our knowledge, this is the first study that focus on the effect of EEDK on corneal Na^+^/K^+^-ATPase. Considering that Na^+^/K^+^-ATPase dysfunction is known to be the root cause of corneal edema, we can propose EEDK to be an effective material for corneal decompensation.

## Data Availability

The authors confirm that the data supporting the findings of this study are available within the article.
